# Exercise Attenuates Skeletal Muscle Atrophy in Senescent SAMP8 Mice: Metabolic Insights from NMR-Based Metabolomics

**DOI:** 10.3390/molecules30092003

**Published:** 2025-04-30

**Authors:** Wenfang Wu, Linglin Zhang, Yifen Chen, Caihua Huang, Longhe Yang, Donghai Lin

**Affiliations:** 1 Key Laboratory for Chemical Biology of Fujian Province, High-Field NMR Center, College of Chemistry and Chemical Engineering, Xiamen University, Xiamen 361005, China; 36520221151882@stu.xmu.edu.cn (W.W.); frost@stu.xmu.edu.cn (L.Z.); cyf1303280@163.com (Y.C.); 2 Research and Communication Center of Exercise and Health, Xiamen University of Technology, Xiamen 361021, China; huangcaihua@xmut.edu.cn; 3 Technical Innovation Center for Utilization of Marine Biological Resources, Third Institute of Oceanography, Ministry of Natural Resources, Xiamen 361021, China

**Keywords:** exercise, aging, skeletal muscle, NMR-based metabolomics, SAMP8

## Abstract

Age-related skeletal muscle atrophy is a major health concern in the elderly, contributing to reduced mobility, increased risk of falls, and metabolic dysfunction. The senescence-accelerated prone 8 (SAMP8) mouse model, known for its rapid aging and early cognitive decline, serves as an essential model for studying age-related muscle degeneration. While previous studies have shown that exercise attenuates muscle atrophy by promoting regeneration and improving strength, the underlying metabolic mechanisms remain poorly understood. This study used the SAMP8 model to evaluate the effects of exercise on muscle atrophy and associated metabolic changes. Our results show that exercise promoted muscle growth by reducing body weight, increasing skeletal muscle mass, and decreasing fat accumulation. Furthermore, exercise improved grip strength, muscle tone, and muscle fiber cross-sectional area, thereby preserving muscle functionality. NMR-based metabolomic analysis identified key metabolic pathways modulated by exercise, including glycine, serine, and threonine metabolism; alanine, aspartate, and glutamate metabolism; pyruvate metabolism; and taurine and hypotaurine metabolism. These findings underscore the therapeutic potential of exercise in combating age-related muscle wasting and elucidate the metabolic pathways underlying its benefits.

## 1. Introduction

Age-related skeletal muscle wasting, or sarcopenia, is a major threat to the health of the elderly. Research suggests that muscle mass declines at a rate of approximately 1% to 2% per year with age [1]. Specifically, 30% of people over the age of 60 have significant muscle wasting, with this prevalence increasing to 50–60% in people over the age of 80 [2]. The consequences of muscle wasting go beyond physical frailty and affect metabolic function. Reduced muscle mass decreases mobility, increases susceptibility to falls, and decreases basal metabolic rate, increasing fat accumulation and risk of metabolic syndrome [3]. In addition, in older people with chronic diseases, muscle wasting exacerbates disease progression and impedes recovery. For example, patients with cardiovascular disease experience reduced cardiac rehabilitation potential due to muscle wasting [4], while cancer patients undergoing chemotherapy suffer from increased fatigue and weakened immune responses, negatively impacting treatment outcomes [5].

The senescence-accelerated prone 8 (SAMP8) mouse model is widely used in aging research due to its accelerated cognitive decline, which closely mimics human neurodegeneration. Transcriptomic analysis of SAMP8 mice reveals alterations in neurogenesis, synaptic plasticity, neuro-metabolism and neuro-inflammation [6]. Biochemical abnormalities in these mice, such as mitochondrial dysfunction [7], oxidative stress [8], and cholinergic neurotransmission deficits, parallel those observed in human aging. Consequently, SAMP8 mice serve as a model to study age-related inflammation, renal injury [9], reproductive aging [10], and cardiac degeneration [11]. Their accelerated muscle senescence has also been a focus for the study of age-related skeletal muscle changes. Studies by Nishikawa et al. [12] and Derave et al. [13] highlight structural muscle changes, reinforcing the utility of the SAMP8 model in sarcopenia research. Guo et al. [14] further validated its relevance by assessing muscle mass, structure, and function.

Exercise has long been recognized as an effective intervention against muscle wasting, acting through multiple molecular pathways. It modulates skeletal muscle protein metabolism by influencing key signaling pathways such as mTOR [15], TGF-β [16], and AMPK-FOXO3a [17], thereby enhancing protein synthesis and inhibiting proteolytic pathways, including the ubiquitin–proteasome [18] and autophagy–lysosome systems [17]. Exercise also attenuates inflammatory responses [19], oxidative stress [20], and apoptosis [21], collectively slowing muscle degeneration. In addition, prolonged aerobic exercise increases systemic longevity by improving rhythmic gene expression in neural and muscle tissues [22]. Recent studies also suggest that exercise can reverse age-related epigenetic modifications and delay skeletal muscle aging.

Despite extensive research on exercise and sarcopenia, few studies have investigated the metabolomic underpinnings of exercise-induced muscle preservation, particularly using nuclear magnetic resonance (NMR) spectroscopy. Given NMR’s superior reproducibility, non-destructive sampling, and high precision in metabolic profiling, it is an ideal tool for elucidating exercise-associated metabolic changes. Here, we use the SAMP8 model and an NMR-based metabolomic approach to dissect the metabolic pathways by which exercise alleviates skeletal muscle atrophy. These findings provide novel insights into the metabolic characteristics of skeletal muscle in the SAMP8 mouse model and contribute to the theoretical framework supporting exercise as an anti-aging intervention.

## 2. Results

**Figure 1:**
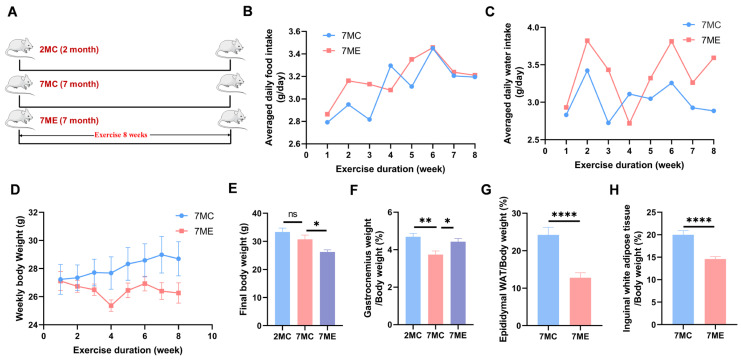
Effects of exercise on growth performance in SAMP8 mice. (**A**) Schematic representation of the experimental design for the 2MC (2-month-old control, young control mice, *n* = 15), 7MC (*n* = 16), and 7ME (*n* = 15) groups. (**B**) Average daily food intake per mouse over the 8-week exercise period in the 7ME (7-month-old exercised, aging mice with exercise) and 7MC (7-month-old control, aging control mice) groups. (**C**) Average daily water intake per mouse during the 8-week exercise period in the 7ME and 7MC groups. (**D**) Weekly body weight changes in the 7ME and 7MC groups during the exercise period. (**E**) Final body weight comparisons among the 2MC, 7MC, and 7ME groups. (**F**) Gastrocnemius muscle weight as a percentage of body weight in the 2MC, 7MC, and 7ME groups. (**G**) Epididymal white adipose tissue (WAT) weight as a percentage of body weight in the 7ME and 7MC groups. (**H**) Inguinal WAT weight as a percentage of body weight in the 7ME and 7MC groups. Statistical significance: ns (not significant, *p* > 0.05); * *p* < 0.05; ** *p* < 0.01; **** *p* < 0.0001.

We used female SAMP8 mice as an aging model to investigate the ameliorative effects of exercise intervention on age-related skeletal muscle atrophy. The SAMP8 mice were divided into three groups: the 2MC (young control) group, which consisted of 2-month-old young mice at the start of the experiment; the 7MC (aging control) group, which consisted of 7-month-old aged mice at the start of the experiment; and the 7ME (aging mice with exercise) group, which underwent an 8-week low-intensity aerobic exercise intervention starting at 7 months of age (Figure 1A). A comprehensive analysis of mouse phenotypes, skeletal muscle metabolism, and metabolic pathways was performed to elucidate the metabolic mechanisms and changes underlying the alleviation of skeletal muscle atrophy in aged mice by exercise intervention.

### 2.1. Exercise Improved Growth Performance in SAMP8 Mice

We found that the exercise intervention had no effect on food or water intake in the aged mice (Figure 1B,C). In addition, the 7MC group showed no change in body weight (Figure 1D,E) but a decrease in skeletal muscle mass (Figure 1F) compared with the 2MC group. In contrast, the 7ME group showed a decrease in body weight, an increase in skeletal muscle mass, and a decrease in both epididymal white adipose tissue (WAT) weight (Figure 1G) and inguinal WAT weight (Figure 1H) compared to the 7MC group.

### 2.2. Exercise Enhanced the Strength and Muscle Fiber Area of Mouse Gastrocnemius Muscle

**Figure 2:**
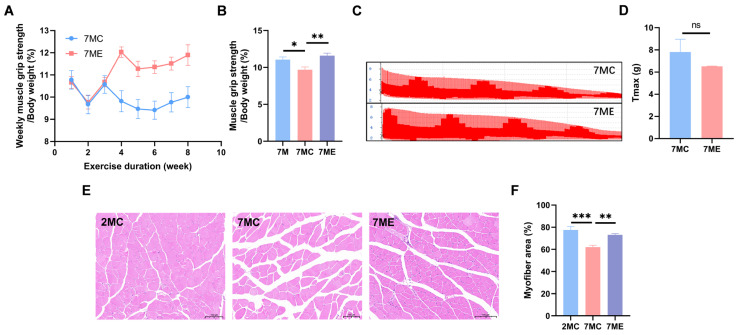
Effects of exercise on skeletal muscle function in SAMP8 mice. (**A**) Weekly progression of muscle grip strength as a percentage of body weight in the 7MC and 7ME groups. (**B**) Comparison of muscle grip strength as a percentage of body weight in mice at 7 months (baseline), 9 months without exercise (7MC), and 9 months with exercise (7ME). (**C**) Representative muscle contraction force profiles in 7ME and 7MC groups. (**D**) Maximum contraction force (T_max_) in the 7MC (*n* = 3) and 7ME (*n* = 3) groups. (**E**) Hematoxylin and eosin-stained gastrocnemius muscle fiber cross-sections in the 2MC (*n* = 3), 7MC (*n* = 3), and 7ME (*n* = 3) groups. (**F**) Quantification of gastrocnemius muscle fiber cross-sectional area in the 2MC, 7MC, and 7ME groups. Statistical significance: ns (not significant, *p* > 0.05); * *p* < 0.05; ** *p* < 0.01; *** *p* < 0.001.

We characterized the grip strength of the mouse gastrocnemius muscle. The results showed that the 7MC group had a decrease in grip strength compared to the 7-month-old aged mice (7M) before exercise intervention. However, after exercise intervention, the grip strength of the 7ME group increased significantly compared to the 7MC group (Figure 2A,B). In addition, exercise intervention improved muscle tone (Figure 2C,D) and increased muscle fiber cross-sectional area (Figure 2E,F) in the mice, indicating that exercise helped mitigate the age-related decline in gastrocnemius muscle mass and strength.

### 2.3. Exercise Induced Changes in the Metabolic Profile of Mouse Gastrocnemius Muscle

**Figure 3:**
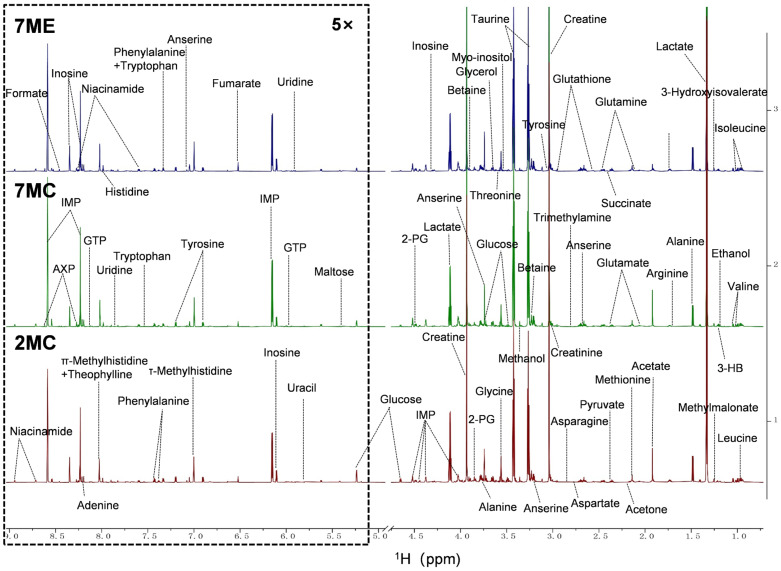
Representative 1D ^1^H-NMR spectra of aqueous metabolites extracted from mouse gastrocnemius muscle. The spectra were recorded on an 850 MHz NMR spectrometer (pH 7.4, 298 K). Vertical scaling was kept constant for all groups, and the resonance region corresponding to water (δ 4.75–5.00 ppm) was removed for clarity. For better visualization, the spectral region spanning δ 5.00–9.00 ppm was magnified five times compared to the 0.75–4.75 ppm region. The spectra for the 2MC (*n* = 15), 7MC (*n* = 16), and 7ME (*n* = 15) groups are shown in red, green, and blue, respectively. Abbreviations: 3-HB, 3-hydroxybutyric acid; 2-PG, 2-phosphoglyceric acid; IMP, inosine monophosphate; GTP, guanosine triphosphate; AXP, adenosine mono/di/triphosphate.

To investigate the effects of exercise intervention on gastrocnemius metabolism of the muscle in aging mice, we analyzed the metabolic profiles of the 2MC, 7MC, and 7ME gastrocnemius muscle groups using 1D ^1^H NMR spectroscopy (Figure 3). A total of 46 metabolites were identified from the NMR spectra (Table S1), including 21 amino acids and their derivatives (leucine, isoleucine, valine, alanine, lysine, arginine, methionine, glutamate, glutamine, glutathione, anserine, aspartate, asparagine, glycine, threonine, tyrosine, phenylalanine, histidine, methylhistidine, creatine, creatinine); 3 sugars and their derivatives (glucose, myo-inositol, 2-phosphoglycerate); 10 organic acids (3-hydroxybutyrate, 3-hydroxyisovalerate, methylmalonate, acetate, lactate, succinate, fumarate, formate, acetone, pyruvate); 1 alcohol (glycerol); and 11 nitrogen-containing metabolites (betaine, taurine, inosine, IMP, inosine, uracil, uridine, adenine, AXP, niacinamide, tryptophan, and GTP).

The resonance assignments of these metabolites were confirmed by two-dimensional nuclear magnetic resonance (NMR) spectroscopy, including 2D ^1^H-^13^C HSQC (Figure S1) and 2D ^1^H-^1^H TOCSY (Figure S2) to ensure the accuracy of the metabolic profile analysis.

**Figure 4:**
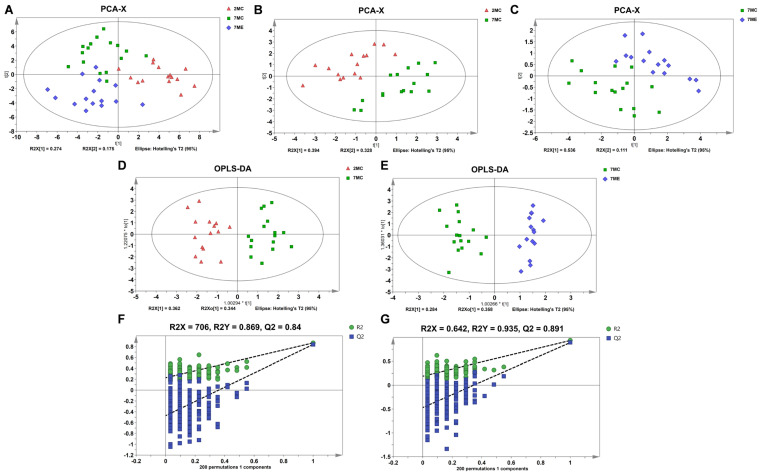
Multivariate statistical analysis of the NMR dataset across three groups of mouse gastrocnemius muscle. (**A**) PCA score plot metabolically comparing the 2MC, 7MC, and 7ME groups. (**B**,**C**) PCA score plots illustrating metabolic differences between the 2MC and 7MC groups (**B**) and between the 7MC and 7ME groups (**C**). (**D**,**E**) OPLS−DA score plots for 2MC vs. 7MC (**D**) and 7MC vs. 7ME (**E**), highlighting distinct metabolic clustering. (**F**,**G**) Cross-validation plots of the OPLS−DA models for 2MC vs. 7MC (F, R^2^_Y_ (cum) = 0.869, Q^2^_Y_ (cum) = 0.84) and 7MC vs. 7ME (G, R^2^_Y_ (cum) = 0.935, Q^2^_Y_ (cum) = 0.891). The reliability of the OPLS−DA models was assessed using a 200−iteration random permutation test.

Unsupervised principal component analysis (PCA) was performed to assess the metabolic characteristics of the different gastrocnemius muscle groups. PCA score plots for the 2MC, 7MC, and 7ME groups revealed distinct clustering patterns among the experimental groups subjected to the exercise intervention (Figure 4A). In addition, pairwise PCA score plots comparing the 2MC group with the 7MC group and the 7ME group with the 7MC group revealed significant metabolic differences between these groups (Figure 4B,C).

To further improve the differentiation of metabolic characteristics between groups, supervised orthogonal partial least squares discriminant analysis (OPLS-DA) was performed based on relative metabolite concentrations. The OPLS-DA score plots clearly showed distinct metabolic separations between the 7MC and 2MC groups (Figure 4D), as well as between the 7ME and 7MC groups (Figure 4E). The robustness and reliability of the OPLS-DA model was confirmed through 200-cycle permutation testing and cross-validation analysis of variance (CV-ANOVA) (Figure 4F,G). These results suggest that both aging and exercise intervention significantly alter the metabolic characteristics of the gastrocnemius muscle in SAMP8 mice.

### 2.4. Exercise Intervention Altered the Levels of Aqueous Metabolites in Mouse Gastrocnemius Muscle

To compare the relative metabolite levels in the three gastrocnemius muscle groups, one-way analysis of variance (ANOVA) was performed on the relative concentrations of 46 metabolites. Tukey’s post hoc multiple comparison test with a significance threshold of *p* < 0.05 was then used to identify metabolites showing significant differences (Table 1).

Compared to the 2MC group, we identified 28 different metabolites in the 7MC group, including 9 upregulated metabolites (3-hydroxybutyrate, succinate, aspartate, creatine, taurine, IMP, uracil, GTP, and AXP) and 19 downregulated metabolites (methylmalonate, alanine, lysine, arginine, methionine, glutamine, acetone, pyruvate, betaine, threonine, glycerol, 2-phosphoglycerate, creatinine, lactate, myo-inositol, glucose, inosine, phenylalanine, and uridine). Compared to the 7MC group, we identified 18 different metabolites in the 7ME group, including 11 upregulated metabolites (alanine, lysine, glutamine, creatine, lactate, inosine, fumarate, niacinamide, uridine, histidine, and AXP) and 7 downregulated metabolites (3-hydroxybutyrate, acetate, succinate, aspartate, asparagine, uracil, and formate).

These results suggest that exercise intervention significantly alters the metabolic profile of the gastrocnemius muscle, affecting a range of metabolites associated with energy metabolism and amino acid metabolism.

### 2.5. Identification of Characteristic Metabolites in Pairwise Comparisons Between Groups

**Figure 5:**
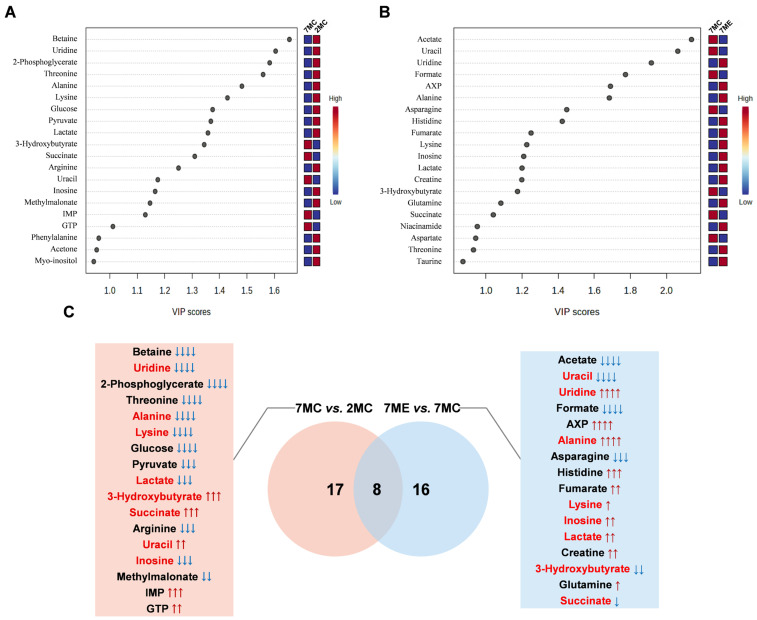
Identification of significant and characteristic metabolites from pairwise comparisons between groups. (**A**,**B**) VIP score ranking plots for significant metabolites identified from the OPLS-DA models comparing 7MC vs. 2MC (**A**) and 7ME vs. 7MC (**B**). (**C**) Venn diagram illustrating characteristic metabolites identified from pairwise comparisons between the three groups. Metabolites highlighted in red are shared between the two comparisons. Metabolites are ranked in descending order based on their VIP scores. Statistical significance: ns (not significant, *p* > 0.05); ↑/↓, *p* < 0.05; ↑↑/↓↓, *p* < 0.01; ↑↑↑/↓↓↓, *p* < 0.001; ↑↑↑↑/↓↓↓↓, *p* < 0.0001.

Using the established OPLS-DA model, we identified key metabolites based on VIP scores > 1. In the OPLS-DA model, a total of 17 key metabolites were identified for the comparison between 7MC and 2MC groups (Figure 5A), while 16 key metabolites were identified for the comparison between 7ME and 7MC groups (Figure 5B).

In the comparison between 7MC and 2MC, 17 characteristic metabolites were identified, whereas 16 characteristic metabolites were identified in the comparison between 7ME and 7MC. Notably, there were eight characteristic metabolites common to both pairwise comparisons (Figure 5C), namely, uridine, uracil, alanine, lysine, lactate, inosine, 3-hydroxybutyrate, and succinate. These eight common characteristic metabolites exhibited contrasting directionality when comparing aging-related decline (7MC vs. 2MC) and exercise-associated restoration (7ME vs. 7MC), while in some cases, exercise mitigated but did not fully reverse age-associated changes.

The levels of several metabolites, including uridine, alanine, lysine, lactate, and inosine, were found to increase after exercise. This suggests that these metabolites may play a critical role in the exercise-induced attenuation of skeletal muscle atrophy. These results highlight significant metabolic shifts between the aging and young groups, as well as the restorative effects of exercise intervention.

### 2.6. Metabolic Pathways Altered by Exercise Intervention

**Figure 6:**
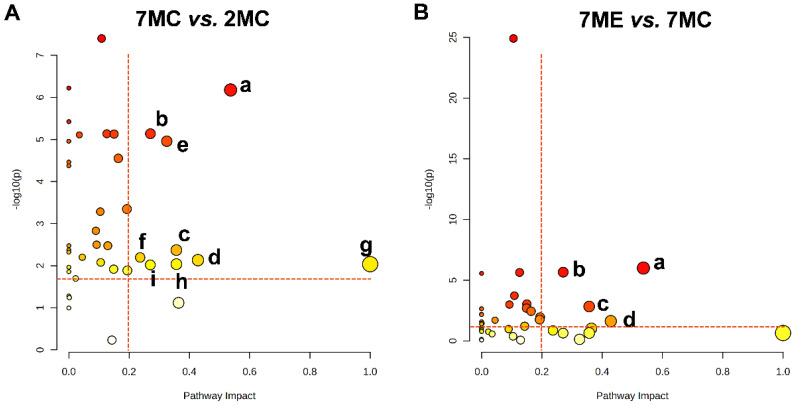
Metabolic pathway analysis from pairwise comparisons between groups based on relative concentrations of metabolites in mouse gastrocnemius muscle. Based on the pathway impact value (PIV > 0.2) and statistical significance (*p* < 0.05) − with the red vertical dashed line indicating PIV > 0.2 and the red horizontal dashed line representing *p* < 0.05 as shown in the figure, the following significantly altered pathways were identified: (**A**) 7MC vs. 2MC: a. alanine, aspartate, and glutamate metabolism; b. pyruvate metabolism; c. glycine, serine, and threonine metabolism; d. taurine and hypotaurine metabolism; e. starch and sucrose metabolism; f. glycerolipid metabolism; g. biosynthesis of phenylalanine, tyrosine, and tryptophan; h. phenylalanine metabolism; i. histidine metabolism. (**B**) 7ME vs. 7MC: a. alanine, aspartate, and glutamate metabolism; b. pyruvate metabolism; c. glycine, serine, and threonine metabolism; d. taurine and hypotaurine metabolism. Larger and darker red circles indicate more significant metabolic pathway alterations, while yellow circles represent pathways with lower statistical significance.

Pathway analysis was performed using a PIV > 0.2 and *p*-value < 0.05 as the criteria. This analysis identified nine significant pathways affected in the comparison between the 7MC and 2MC groups (Figure 6A), and four significant pathways affected in the comparison between the 7ME and 7MC groups (Figure 6B). Four key pathways were found to be common to both comparisons, including pyruvate metabolism, glycine, serine, and threonine metabolism; alanine, aspartate, and glutamate metabolism; and taurine and hypotaurine metabolism (Table S2).

These results suggest that exercise intervention in aging mice primarily affects amino acid metabolism, oxidative stress pathways, and energy metabolism, which contribute to the attenuation of skeletal muscle atrophy.

**Figure 7:**
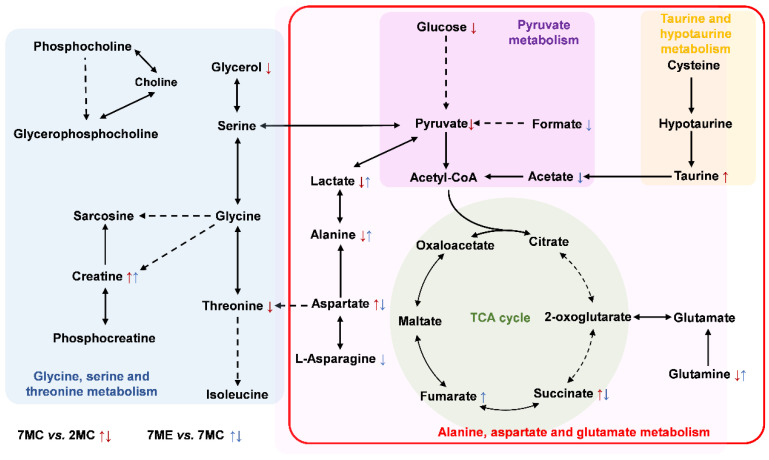
Metabolic alterations in gastrocnemius muscle from pairwise comparisons between groups. Key metabolic pathways affected include glycine, serine, and threonine metabolism (blue box); pyruvate metabolism (purple box); taurine and hypotaurine metabolism (yellow box); and alanine, aspartate, and glutamate metabolism (red box). The TCA cycle (green dashed circle) is also included to highlight interconnected energy metabolism pathways. Dotted arrows represent multi-step reactions, while solid arrows indicate one-step reactions. Metabolic changes are indicated as follows: ↑/↓ in red—significant differences between the 7MC and 2MC groups; ↑/↓ in blue—significant differences between the 7ME and 7MC groups.

To visually illustrate the changes in the characteristic metabolites across the three groups, we projected these metabolites onto a metabolic map based on the Kyoto Encyclopedia of Genes and Genomes (KEGG) database (Figure 7). The changes in characteristic metabolites and significant changes in metabolic pathways provide new insights into the molecular mechanisms underlying the effects of exercise intervention on the gastrocnemius muscle of aging mice.

## 3. Discussion

Age-related muscle wasting is a critical issue for the elderly population as it significantly impacts physical function, mobility, and overall quality of life. The loss of muscle mass and strength, known as sarcopenia, is a major contributor to frailty and disability in older adults. Previous research has shown that exercise can effectively combat skeletal muscle wasting by promoting muscle regeneration and improving strength. Regular lifelong physical activity maintains enhanced cardiorespiratory fitness, muscle mass, and metabolic homeostasis throughout life, thereby establishing a foundation for healthy aging [23,24]. In contrast, late-life exercise initiation (i.e., commencing structured physical activity in older age) may not fully reverse age-associated physiological deterioration, but it can yield clinically meaningful improvements in functional capacity, mitigate the progression of sarcopenia, and reduce age-related disease risks—highlighting its practical relevance and translational value for geriatric populations [25,26]. Therefore, in this study, we used the SAMP8 mouse model, a well-established model of accelerated aging, to investigate the effects of exercise intervention on age-related skeletal muscle atrophy. Specifically, we aimed to evaluate the efficacy of exercise in attenuating age-related muscle loss and to explore the underlying metabolic pathways that may contribute to this beneficial effect. By combining advanced techniques such as NMR-based metabolomics, our study aims to uncover the molecular mechanisms by which exercise may counteract the metabolic changes associated with muscle atrophy in aging.

In this study, exercise intervention improved growth performance in mice by reducing body weight, increasing skeletal muscle mass, and decreasing both epididymal fat and white adipose tissue mass. In addition, exercise ameliorated age-related declines in gastrocnemius muscle strength and function by improving grip strength, muscle tone, and muscle fiber cross-sectional area.

To explore the underlying metabolic mechanisms, we performed NMR-based metabolomic analysis. The results showed that the levels of several metabolites, including uridine, alanine, lysine, lactate, and inosine, were increased after exercise. These findings suggest that these metabolites may be involved in the exercise-associated attenuation of skeletal muscle atrophy. Furthermore, exercise intervention was accompanied by alterations in several key metabolic pathways that may be linked to the observed attenuation of muscle atrophy, including glutamate, serine, and threonine metabolism; alanine, aspartate, and glutamate metabolism; pyruvate metabolism; and taurine and hypotaurine metabolism. These findings suggest that these metabolites may play a critical role in the exercise-induced attenuation of age-related skeletal muscle atrophy and may serve as potential biomarkers for the efficacy of exercise in combating aging. Furthermore, exercise intervention was found to slow the progression of muscle atrophy by modulating key metabolic pathways, including glutamate, serine, and threonine metabolism; alanine, aspartate, and glutamate metabolism; pyruvate metabolism; and taurine and hypotaurine metabolism.

### 3.1. Exercise Modulated Glycine, Serine, and Threonine Metabolism

Glycine, serine, and threonine are essential amino acids vital for skeletal muscle health. Besides their role in protein synthesis, they serve as precursors for phospholipids, sphingolipids, and neurotransmitters, supporting structural integrity and metabolic balance. Aging disrupts their metabolism, leading to reduced levels in muscle and contributing to impaired protein synthesis, mitochondrial dysfunction, and muscle decline [27,28].

In our study, aging mice exhibited significantly lower levels of threonine and glycerol-related metabolites in skeletal muscle compared to young controls, while glycine levels remained largely unchanged. Notably, in comparison to young mice, creatine levels were elevated in aging muscle and showed a further increase after exercise. However, exercise failed to restore threonine or glycerol metabolite levels. These findings suggest that aging impairs both amino acid and energy metabolism in skeletal muscle, and that some of these alterations may be resistant to short-term exercise interventions.

Threonine, a key component of the glycine–serine–threonine metabolic axis, plays a critical role in one-carbon metabolism [29], nucleotide biosynthesis [30], and mitochondrial function [31]. The age-related decrease in threonine may reflect impaired anabolic activity and reduced mitochondrial efficiency, both characteristic features of sarcopenia. The failure to restore threonine levels after exercise suggests that threonine metabolism in aging muscle may be less adaptable to exercise-induced metabolic remodeling, possibly due to persistent deficits in amino acid transport or enzymatic activity [32,33]. Similarly, the decline in glycerol metabolites in aging skeletal muscle may be attributed to altered lipid mobilization or impaired glycolytic flux. As a central intermediate in triglyceride metabolism and gluconeogenesis, reduced glycerol levels may indicate diminished energy availability or increased metabolic inflexibility with aging [34].

In contrast, the progressive increase in creatine levels observed with aging and further enhanced by exercise may reflect an adaptive response to elevated energy demands in skeletal muscle. As a readily mobilizable phosphate donor, creatine plays a critical role in buffering ATP levels during periods of high muscular activity [35]. Its accumulation in aged muscle likely represents a compensatory upregulation of the phosphocreatine system in response to declining mitochondrial ATP production [36]. The additional rise in creatine following exercise may indicate an amplified metabolic adaptation aimed at supporting the heightened energy requirements associated with physical activity.

### 3.2. Exercise Modulated Alanine, Aspartate, and Glutamate Metabolism

Alanine, aspartate, and glutamate are central to skeletal muscle metabolism, supporting energy production, amino acid turnover, and nitrogen balance through their involvement in key metabolic pathways [37,38,39]. In this study, compared to young mice, aged mice exhibited a significant reduction in alanine levels, a moderate increase in aspartate, and stable glutamate levels. Following exercise intervention, alanine levels showed a partial recovery, while aspartate exhibited a modest decrease. These results suggest that aging selectively disrupts amino acid metabolism in skeletal muscle, and that some of these alterations may be partially mitigated through exercise.

The decline in alanine with aging may reflect reduced glycolytic flux or impaired transaminase activity, both characteristic of mitochondrial dysfunction and metabolic inflexibility [40]. The partial restoration of alanine levels following exercise suggests a modest reactivation of glycolysis or transamination, indicating some recovery of metabolic plasticity.

Aspartate plays key roles in the malate–aspartate shuttle, nucleotide biosynthesis, and the urea cycle. Its mild accumulation in aging muscle may reflect altered mitochondrial metabolism or decreased biosynthetic utilization [41], while the slight decline after exercise could suggest increased mitochondrial flux or nucleotide turnover during muscle remodeling.

In contrast, glutamate levels remained unchanged across all groups. As a central metabolite in nitrogen balance, transamination, and TCA cycle anaplerosis, its stability may point to preserved regulatory control, even under conditions of metabolic stress [42].

The concurrent rise in fumarate—a TCA cycle intermediate—after exercise further supports enhanced mitochondrial activity and ATP production in aged muscle [43]. These findings highlight the ability of exercise to regulate alanine, aspartate, and glutamate metabolism and ultimately mitigate age-related muscle wasting by restoring metabolic homeostasis and improving mitochondrial function.

### 3.3. Exercise Modulated Pyruvate Metabolism

Pyruvate is a critical metabolic intermediate that links glycolysis and the tricarboxylic acid (TCA) cycle, both of which are essential for energy production to support muscle function. As individuals age, there is a significant decrease in glucose utilization and pyruvate production in skeletal muscle tissue. This decline impairs the glycolytic pathway, resulting in decreased energy output and contributing to muscle wasting [44].

Exercise has been shown to reverse or ameliorate many age-related metabolic deficiencies, particularly those involving pyruvate metabolism. Regular physical activity increases glycolytic activity by increasing glucose uptake and glycolysis within muscle fibers. Exercise stimulates key enzymes in the glycolytic pathway, resulting in an increase in the conversion of glucose to pyruvate. This process not only increases the pyruvate pool, but also improves the efficiency of the TCA cycle and oxidative phosphorylation in muscle mitochondria [45]. More specifically, exercise has been shown to activate the AMPK pathway, which promotes the expression of enzymes involved in glycolysis, which in turn increases pyruvate production. This in turn supports mitochondrial function, ATP generation, and overall muscle health [46].

Metabolites such as formate and acetate are byproducts of alternative pathways that become more pronounced when glycolytic efficiency is impaired. A decrease in formate and acetate levels indicates a shift to more efficient aerobic metabolism where pyruvate is fully oxidized in the mitochondria. This transition improves muscle endurance by reducing reliance on anaerobic pathways, which are less efficient at producing energy and contribute to muscle fatigue. These findings suggest that exercise helps to optimize energy metabolism by reducing reliance on these byproducts, promoting more efficient oxidative pathways, and ultimately supporting improved energy production and muscle function.

### 3.4. Exercise Modulated Taurine and Hypotaurine Metabolism

Taurine plays multiple roles in skeletal muscle metabolism, including regulating calcium homeostasis [47], acting as an antioxidant, stabilizing cell membranes [48], modulating energy metabolism, exerting anti-inflammatory effects [49], and promoting muscle repair and regeneration. These functions make taurine an essential molecule for maintaining skeletal muscle health and function. The potential applications of taurine in exercise science and clinical medicine are being studied extensively, particularly in improving exercise performance, delaying muscle aging, and treating metabolic diseases. Taurine, a sulfur-containing amino acid, plays a critical role in maintaining skeletal muscle function and overall metabolic health. Studies have shown that taurine is critical in regulating calcium ion balance, providing antioxidant defense, stabilizing membranes, and modulating energy metabolism. These functions are essential for muscle contraction, reducing oxidative stress and preventing muscle damage.

In this study, we observed a significant increase in taurine levels in skeletal muscles of aging mice, suggesting that taurine homeostasis and its metabolic pathways may be disrupted during the aging process. Despite the increased taurine levels in aging, this increase was not sufficient to fully counteract the muscle atrophy induced by oxidative stress and metabolic dysfunction. This phenomenon suggests that aging is associated not only with changes in taurine concentration, but also with disturbances in its metabolic pathways. Exercise as an intervention has been shown to effectively regulate taurine metabolism and, in turn, mitigate or reverse age-related skeletal muscle atrophy. Previous studies suggest that exercise activates key enzymes and pathways that increase taurine utilization in muscle cells [50]. This not only helps stabilize taurine levels but also promotes muscle cell repair and regeneration.

## 4. Materials and Methods

### 4.1. Animal Experiments

All procedures involving animals were carefully performed in accordance with the ethical standards approved by the Experimental Animal Ethics Committee of the Third Institute of Oceanography, Ministry of Natural Resources (license number: TIO-IACUC-03-2024-3-01), ensuring strict adherence to guidelines for the humane treatment and welfare of laboratory animals. The living conditions of the mice were strictly controlled, with a 12 h light/dark cycle, a stable ambient temperature of 23 *±* 3 °C, and a relative humidity of 70 *±* 5%. After one week of acclimatization, female SAMP8 mice were systematically divided into three different groups: the young control group (2MC, *n* = 15), the aging control group (7MC, *n* = 16), and the aging mice with exercise (7ME, *n* = 15) group.

At the beginning of the experiment, young control (2MC) group mice were used together with February aged SAMP8 mice. Since the literature indicates that skeletal muscle atrophy begins to develop in July aged [51,52], these mice were used for both the aging control (7MC) group and the aging with exercise (7ME) groups. The latter group was acclimated to exercise for one week before being subjected to a moderate-intensity aerobic exercise program for eight weeks (15 m/min, 1 h per day, 5 days per week) [53,54]. This exercise protocol was chosen because of its proven efficacy in reducing skeletal muscle atrophy, as supported by previous studies. In contrast, the young control and aging control groups were fed ad libitum without any intervention. After eight weeks, all three groups of mice were euthanized and sampled.

Throughout the experiment, food consumption, water intake, and body weight were closely monitored. At the end of the eight-week period, comprehensive assessments were performed according to the protocols described in Section 4.2, Section 4.3, Section 4.4. These assessments evaluated skeletal muscle strength and functional capacity, including gastrocnemius muscle contractility and muscle fiber morphology. The results provided valuable insight into the efficacy of the exercise intervention in slowing skeletal muscle atrophy in aging SAMP8 mice.

### 4.2. Grip Strength Test

Grip strength of mouse limbs was measured using a grip strength tester (Ugo Basile 47200 Grip Meter, Gemonio, Italy) after the device was securely installed. The instrument was set to test mode, and each mouse was gently held by the base of the tail and positioned horizontally above the metal grid. The mouse was then lowered until its forelimbs (or all limbs) spontaneously grasped the grid, a natural reflexive behavior in mice. While maintaining a steady horizontal orientation, the tail was gently pulled backward until the mouse released its grip. The peak force at the moment of release was automatically recorded by the instrument. Each mouse was tested three times, and the average of the three measurements was used for subsequent analysis.

All results are expressed as mean ± standard deviation (mean ± SD). Statistical analysis was conducted using one-way ANOVA followed by Tukey’s multiple comparison test to assess differences between groups. A *p*-value < 0.05 was considered statistically significant, with thresholds set at *p* < 0.05 (*), *p* < 0.01 (**), and *p* < 0.001 (***).

### 4.3. Assessment of Gastrocnemius Muscle Contractility

After euthanasia, each gastrocnemius muscle was carefully dissected and immediately immersed in a Petri dish containing Krebs solution adjusted for physiological accuracy. The muscle was then prepared for mechanical testing. One end was attached to a precision transducer with surgical sutures, while the opposite end was attached to a fixed hook at the base of a chamber filled with a specially formulated physiological saline solution. This solution, which contained 10 mM glucose, 2.5 mM CaCl_2_, 10 mM HEPES buffer, 140 mM NaCl, 5 mM KCl, and 2 mM MgCl_2_, was designed to mimic the natural ionic environment of the muscle to ensure accurate contractility measurements.

The time from induction of anesthesia to initiation of muscle testing was consistently maintained at approximately three minutes for each mouse. During this time, the hindlimb was prepared by excising the skin to expose the Achilles tendon, gastrocnemius, and soleus muscles. Electrodes were then attached to the gastrocnemius muscle to initiate contractility testing. After evaluation, humane euthanasia was performed by cervical dislocation. The gastrocnemius muscle was immediately excised and rapidly preserved in liquid nitrogen to ensure uniformity and procedural consistency across experimental groups.

Once stabilized in physiological solution, the contractile properties of the muscle were rigorously assessed, focusing on peak contractile force (T_max_). These metrics were accurately measured using the BL-420F Biosignal Acquisition and Analysis System (Chengdu Taimeng Software Co., Ltd., Chengdu, China). This methodological approach allowed for a thorough evaluation of the functional integrity and resilience of the gastrocnemius muscle in the context of this study [55].

### 4.4. Morphometric Evaluation of Gastrocnemius Muscle Fibers

Gastrocnemius muscle samples were initially fixed in 4% paraformaldehyde and subjected to a 24 h fixation period to preserve structural integrity. After fixation, the samples underwent a rigorous dehydration process through a graded ethanol series and were finally embedded in paraffin matrix to allow accurate sectioning. From these embedded tissues, 5 μm thick sections were carefully prepared and then stained with hematoxylin and eosin (H&E). This traditional histological staining technique distinguishes between cellular and extracellular matrix components, significantly improving the delineation of muscle fiber architecture.

After staining, the sections were carefully mounted and sealed with coverslips to protect their morphologic integrity. High-resolution images of these stained sections were captured using a sophisticated light microscope equipped with digital imaging technology. These images provided the basis for subsequent quantitative morphometric analysis. The cross-sectional area and diameter of individual muscle fibers were determined using ImageJ software (version 1.51j8), a respected image processing tool known for its precision in morphometric evaluations. This digital examination allowed precise measurement of muscle fiber dimensions and provided a crucial understanding of the morphological changes in the gastrocnemius muscle under different experimental conditions.

### 4.5. Preparation of Gastrocnemius Muscle Samples for NMR Experiments

The preparation of gastrocnemius muscle samples for NMR spectroscopic analysis was performed meticulously according to well-established protocols cited in the literature [56,57]. Upon completion of the animal experiments, the mice were first anesthetized with phenobarbital and then humanely euthanized by cervical dislocation. Immediately after euthanasia, the skin of the hind limbs was gently peeled back to expose the Achilles, gastrocnemius, and soleus muscles. These muscles were quickly dissected from the limbs and placed in sterile 1.5 mL tubes. To immediately halt tissue metabolism and inhibit enzymatic activity, the dissected samples were immersed in liquid nitrogen within seconds of dissection to ensure the preservation of their biochemical integrity for subsequent NMR analysis. Each NMR sample was prepared from the gastrocnemius muscle of a single mouse, with no pooling of tissue across individuals. The sample sizes were as follows: 2MC (*n* = 15), 7MC (*n* = 16), and 7ME (*n* = 15). Thus, each NMR spectrum corresponds to a distinct biological replicate, allowing for robust statistical analysis and accurate representation of inter-individual variability.

To homogenize the tissue samples, approximately 100 mg of gastrocnemius muscle was weighed and transferred to a homogenization tube. Pre-cooled methanol and water at a ratio of 1:0.95 (*w*/*v* = 1:10) were added to the tube along with grinding steel beads. The sample was then homogenized using a 65 Hz grinder at a temperature of 4 °C for 60 s. After this initial grinding, pre-cooled chloroform at a ratio of *w*/*v* = 1:10 was added and the homogenate was processed again under the same conditions. The primary functions of methanol and chloroform were to quench protein activity and to facilitate the extraction of water-soluble and lipid-soluble metabolites [57], respectively. Our focus was on water-soluble metabolites; therefore, after homogenization, the mixture was centrifuged to separate the phases, and only the aqueous phase was retained for further analysis. After evaporating the methanol, any remaining water was removed by lyophilization, resulting in a dry powder of the extracted metabolites.

The concentrated metabolite extracts were reconstituted in 550 μL of NMR buffer composed of deuterium oxide (D_2_O) containing 50 mM phosphate buffer (PBS, pH 7.4) and 1 mM trimethylsilylpropanoic acid (TSP). After thorough mixing, samples were centrifuged at 12,000× *g* for 10 min to remove any residual particulates, and the supernatant was transferred to a 5 mm NMR tube for spectral acquisition.

A 0.5 M PBS stock solution was prepared by dissolving 5.7 g of K_2_HPO_4_·3H_2_O and 3.9 g of NaH_2_PO_4_·2H_2_O in 50 mL of D_2_O. The pH was carefully adjusted to 7.4 using a calibrated pH meter and NaOH solution. The final NMR buffer ensured stable sample pH during acquisition, which is critical for chemical shift consistency across samples. D_2_O served as the deuterium lock signal to stabilize the magnetic field, and TSP was used as an internal standard for chemical shift referencing and metabolite quantification.

### 4.6. NMR Experiments

All NMR experiments were performed at 298 K using a high-resolution Bruker Avance III HD 850 MHz spectrometer (Bruker, Germany) equipped with a TCI cryogenic probe for enhanced detection sensitivity. One-dimensional (1D) ^1^H NMR spectra were recorded using the standard NOESYGPPR1D pulse sequence, designated [RD-G_1_-90°-τ_m_-G_2_-90°-ACQ], which was specifically designed for effective water suppression [58]. This was achieved by suppressing the water signal during both the relaxation delay (RD) and the mixing time (τ_m_), with the inclusion of pulse gradients G_1_ and G_2_ to further enhance the attenuation of the water peak. The main experimental parameters for the 1D ^1^H NMR spectra were as follows: τ_m_ = 10 ms; ACQ = 2.66 s; RD = 4 s. A total of 64 transients were collected into 64 K data points, with a spectral width of 20 ppm [59].

To validate the assignment of metabolite signals in the NMR spectra, two-dimensional (2D) ^1^H–^13^C heteronuclear single quantum coherence (HSQC) spectra were acquired with the pulse sequence hsqcetgpsisp2.2 [60]. The spectral width was set to 10 ppm in the ^1^H dimension and 130 ppm in the ^13^C dimension. The relaxation delay (RD) was 1.5 s, and the data matrix was collected with 1024 × 128 points. Additionally, 2D ^1^H–^1^H total correlation spectroscopy (TOCSY) spectra [60] were acquired with a spectral width of 10 ppm in both dimensions, a relaxation delay of 1.5 s, and a data matrix of 2048 × 256 points. All spectra were recorded at a temperature of 298 K using a Bruker AVANCE III 850 MHz NMR spectrometer (Bruker, Germany).

### 4.7. NMR Spectral Data Processing and Resonance Assignment

The 1D NMR spectral data were initially pre-processed and Fourier transformation using Topspin software (version 4.4.0). This was followed by extensive spectral refinement in MestReNova 9.0 software, including phase correction, baseline adjustment, and chemical shift calibration to ensure accuracy and consistency across all spectra. The chemical shift calibration was carefully anchored to the methyl resonance peak of TSP (δ = 0.00 ppm). The water resonance region (4.75–5.00 ppm) was excluded to eliminate any confounding effects of the water signal on metabolite quantification and multivariate analysis. In addition, the data were normalized relative to the TSP integral and the mass of the gastrocnemius muscle tissue to ensure that the resulting data matrix accurately reflected the relative metabolite concentrations, thereby facilitating robust and meaningful multivariate analysis.

The processed spectra were then superimposed for comprehensive analysis. This overlay facilitated a careful peak alignment process on the combined spectra to ensure consistency in peak positions. The aligned spectra were then segmented into discrete regions with a spectral region (bin) of 0.001 ppm. These quantified data were imported into MATLAB R2015b, where they were organized into a structured data matrix, providing the basis for in-depth multivariate statistical analysis. Resonance assignments of metabolites were performed based on the 1D 1H spectra using a combination of the Chenomx NMR Suite software (version 8.3, Chenomx Inc., Edmonton, AB, Canada), the Human Metabolome Database (HMDB, http://www.hmdb.ca/, accessed on 5 March 2023), and relevant literature.

The 2D raw spectral data were processed using TopSpin 4.0.6 (Bruker BioSpin, Ettlingen, Germany). Zero-filling was applied in both dimensions to double the number of acquired points, enhancing digital resolution. A squared sine-bell apodization function was applied prior to Fourier transformation to improve spectral quality and minimize truncation artifacts. Baseline correction was performed using a polynomial fitting algorithm, and manual phase correction was applied when necessary to ensure optimal spectral clarity. Chemical shift calibration was carefully referenced to the methyl resonance peak of TSP (δ = 0.00 ppm).

### 4.8. Multivariate Statistical Analysis and Significant Metabolite Identification

The NMR dataset was subjected to multivariate statistical analysis using MetaboAnalyst 6.0 (http://www.metaboanalyst.ca, accessed on 4 March 2025) and SIMCA-P+ software (version 14.1) [61]. Pareto scaling was applied to the baseline data to effectively reduce the influence of varying metabolite concentrations on the analytical results. Unsupervised principal component analysis (PCA) was used to examine the metabolic patterns between groups, elucidate potential outliers, and delineate group differences. Supervised partial least squares discriminant analysis (PLS-DA) was then used to improve discrimination between the different metabolic profiles of the groups. The robustness and reliability of the OPLS-DA model was confirmed through 200-cycle permutation testing and cross-validation analysis of variance (CV-ANOVA). Both the calculated explanatory power (R_Y_^2^(cum)) and predictive accuracy (Q_Y_^2^(cum)), which approach one, indicate the high reliability of the OPLS-DA model, demonstrating its strong capacity to explain and predict variance within the dataset.

The PLS-DA model was used to identify significant metabolites based on a criterion of variable importance in projection (VIP) > 1. These metabolites were considered to be important contributors to the observed metabolic differences between groups and therefore merited further investigation for their potential biological relevance.

### 4.9. Univariate Statistical Analysis and Differential Metabolite Identification

Metabolite concentration data were subjected to univariate statistical analysis using SPSS software (version 23.0). Pairwise comparisons between groups were performed using one-way analysis of variance (ANOVA), followed by Tukey’s multiple comparison test to detect statistically significant differences. Differential metabolites between the groups were identified using a criterion of *p* < 0.05. Characteristic metabolites were identified using the dual criteria of *p* < 0.05 and VIP > 1. This approach facilitated the identification of metabolites that were both statistically significant and highly influential in the model, thereby highlighting those most relevant to the observed metabolic differences between the groups.

### 4.10. Metabolic Pathway Analysis and Significant Pathway Identification

Metabolite concentration data were analyzed using the pathway analysis module of MetaboAnalyst 6.0 (https://www.metaboanalyst.ca, accessed on 4 March 2025) to uncover significant pathways affected by changes in metabolite levels. A dual approach was used for this analysis. First, a metabolite set enrichment analysis identified significant pathways with *p* < 0.05, indicating pathways enriched in metabolites that varied significantly between samples, and suggesting their biological relevance. Subsequently, a pathway topological analysis assigned a pathway impact value (PIV), where pathways with PIV > 0.2 were considered significant. This highlighted the essential roles and interactions of metabolites within these pathways, indicating not only statistical significance but also deep biological importance. Pathways that met both criteria (*p* < 0.05 and PIV > 0.2) were considered biologically significant and potentially responsive to experimental conditions.

### 4.11. General Statistical Analyses

Experimental data are presented as mean ± SD, and statistical analysis was performed using IBM SPSS Statistics 22.0 software (IBM, New York, NY, USA). One-way ANOVA followed by Tukey’s multiple comparison test was used to compare the three groups of mouse gastrocnemius. Statistical significance was determined as *p* > 0.05 (no statistical significance, ns), *p* < 0.05 (*), *p* < 0.01 (**), *p* < 0.001 (***), *p* < 0.0001 (****).

## 5. Conclusions

This study highlights the efficacy of exercise in mitigating age-related muscle atrophy as evidenced by improved muscle mass, grip strength, and metabolic homeostasis in SAMP8 mice. Metabolomic analysis highlights key pathways affected by exercise, particularly amino acid metabolism and oxidative stress regulation. These findings support the therapeutic potential of exercise as a non-pharmacological intervention against sarcopenia.

Although our findings suggest an association between exercise-induced metabolic alterations and reduced muscle atrophy, we acknowledge that this study does not establish a direct causal relationship. Additionally, the absence of a wild-type control group limits our ability to determine whether the observed metabolic changes are specific to the SAMP8 aging phenotype or reflect general age-related responses. Future studies incorporating wild-type controls and targeted mechanistic experiments (e.g., metabolite supplementation or inhibition) are warranted to clarify these relationships and further explore potential synergistic effects of exercise and metabolic modulation on muscle preservation during aging.

## Figures and Tables

**Table 1:** Univariate statistical analysis of relative concentrations of aqueous metabolites in mouse gastrocnemius muscle.

Metabolites	Mean ± SD			Multiple Comparisons			One-Way ANOVA	
	2MC	7MC	7ME	7MC vs. 2MC	7ME vs. 7MC	7ME vs. 2MC	F-Value	*p*-Value
Leucine	1.199 ± 0.149	1.072 ± 0.169	0.947 ± 0.223	ns	ns	↓↓	7.156	0.002
Isoleucine	0.402 ± 0.054	0.361 ± 0.062	0.342 ± 0.073	ns	ns	↓	3.489	0.039
Valine	0.603 ± 0.080	0.522 ± 0.099	0.506 ± 0.112	ns	ns	↓	4.28	0.02
3-Hydroxybutyrate	0.152 ± 0.064	0.327 ± 0.124	0.199 ± 0.074	↑↑↑	↓↓	ns	33.071	<0.0001
3-Hydroxyisovalerate	0.150 ± 0.007	0.144 ± 0.009	0.141 ± 0.008	ns	ns	↓	4.94	0.012
Methylmalonate	0.132 ± 0.014	0.108 ± 0.020	0.121 ± 0.024	↓↓	ns	ns	5.887	0.005
Alanine	2.603 ± 0.237	2.130 ± 0.174	2.487 ± 0.168	↓↓↓↓	↑↑↑↑	ns	24.889	<0.0001
Lysine	1.362 ± 0.308	0.885 ± 0.129	1.149 ± 0.285	↓↓↓↓	↑	ns	14.026	<0.0001
Acetate	1.468 ± 0.103	1.401 ± 0.072	0.490 ± 0.078	ns	↓↓↓↓	↓↓↓↓	620.643	<0.0001
Arginine	0.471 ± 0.079	0.360 ± 0.052	0.362 ± 0.089	↓↓↓	ns	↓↓↓	10.924	<0.001
Methionine	0.462 ± 0.046	0.412 ± 0.050	0.400 ± 0.037	↓↓	ns	↓↓	8.169	0.001
Glutamate	0.737 ± 0.090	0.692 ± 0.110	0.679 ± 0.105	ns	ns	ns	1.309	0.281
Glutamine	1.075 ± 0.127	0.948 ± 0.109	1.064 ± 0.101	↓↓	↑	ns	6.119	0.005
Acetone	0.038 ± 0.010	0.028 ± 0.009	0.023 ± 0.007	↓↓	ns	↓↓↓	11.259	<0.001
Pyruvate	0.037 ± 0.017	0.014 ± 0.005	0.016 ± 0.006	↓↓↓	ns	↓↓	19.601	<0.0001
Succinate	0.019 ± 0.007	0.049 ± 0.023	0.031 ± 0.009	↑↑↑	↓	↑↑	23.158	<0.0001
Glutathione	0.332 ± 0.034	0.300 ± 0.054	0.268 ± 0.058	ns	ns	↓↓	6.14	0.005
Anserine	0.783 ± 0.085	0.869 ± 0.090	0.893 ± 0.143	ns	ns	↑	4.29	0.02
Aspartate	0.091 ± 0.026	0.122 ± 0.036	0.093 ± 0.027	↑	↓	ns	5.014	0.011
Asparagine	0.053 ± 0.028	0.081 ± 0.044	0.029 ± 0.008	ns	↓↓↓	↓	26.998	<0.001
Creatine	19.392 ± 1.127	20.484 ± 1.373	22.057 ± 1.169	↑	↑↑	↑↑↑↑	17.763	<0.0001
Betaine	0.628 ± 0.065	0.452 ± 0.049	0.496 ± 0.087	↓↓↓↓	ns	↓↓↓↓	27.575	<0.0001
Taurine	18.223 ± 1.225	19.458 ± 1.162	20.447 ± 1.143	↑	ns	↑↑↑↑	13.458	<0.0001
Glycine	1.085 ± 0.327	0.939 ± 0.110	0.866 ± 0.071	ns	ns	↓	4.58	0.016
Threonine	0.140 ± 0.015	0.097 ± 0.012	0.110 ± 0.018	↓↓↓↓	ns	↓↓↓↓	29.985	<0.0001
Glycerol	1.047 ± 0.086	0.954 ± 0.089	0.895 ± 0.124	↓	ns	↓↓↓	8.603	0.001
2-Phosphoglycerate	1.452 ± 0.106	1.117 ± 0.125	1.086 ± 0.167	↓↓↓↓	ns	↓↓↓↓	34.294	<0.0001
Creatinine	0.037 ± 0.007	0.030 ± 0.005	0.033 ± 0.008	↓	ns	ns	3.914	0.027
Lactate	7.931 ± 0.602	6.285 ± 1.044	7.681 ± 1.249	↓↓↓	↑↑	ns	12.224	<0.0001
Myoinositol	0.141 ± 0.018	0.122 ± 0.015	0.123 ± 0.021	↓	ns	↓	5.374	0.008
IMP	1.205 ± 0.113	1.373 ± 0.111	1.430 ± 0.117	↑↑↑	ns	↑↑↑↑	15.945	<0.0001
Glucose	0.281 ± 0.066	0.154 ± 0.067	0.146 ± 0.081	↓↓↓↓	ns	↓↓↓↓	17.077	<0.0001
Uracil	0.009 ± 0.001	0.011 ± 0.002	0.003 ± 0.001	↑↑	↓↓↓↓	↓↓↓↓	36.899	<0.0001
GTP	0.014 ± 0.002	0.017 ± 0.002	0.016 ± 0.002	↑↑	ns	↑↑↑	6.242	0.004
Inosine	0.250 ± 0.042	0.194 ± 0.031	0.242 ± 0.041	↓↓↓	↑↑	ns	10.1	<0.001
Fumarate	0.050 ± 0.006	0.047 ± 0.006	0.060 ± 0.012	ns	↑↑	↑	28.687	0.001
Tyrosine	0.132 ± 0.016	0.122 ± 0.011	0.112 ± 0.019	ns	ns	↓↓	6.116	0.005
1-Methylhistidine	0.509 ± 0.065	0.557 ± 0.063	0.580 ± 0.101	ns	ns	↑	3.218	0.05
Phenylalanine	0.162 ± 0.019	0.141 ± 0.016	0.136 ± 0.026	↓	ns	↓↓	6.265	0.004
Niacinamide	0.090 ± 0.006	0.089 ± 0.005	0.094 ± 0.006	ns	↑	ns	4.222	0.021
Tryptophan	0.016 ± 0.002	0.016 ± 0.002	0.014 ± 0.002	ns	ns	↓↓↓	3.153	0.053
Uridine	0.013 ± 0.002	0.005 ± 0.002	0.018 ± 0.004	↓↓↓↓	↑↑↑↑	↑↑	28.277	<0.0001
Histidine	0.083 ± 0.014	0.084 ± 0.012	0.107 ± 0.017	ns	↑↑↑	↑↑	14.498	<0.0001
Adenine	0.068 ± 0.012	0.068 ± 0.008	0.064 ± 0.013	ns	ns	↓↓	0.639	0.533
AXP	0.050 ± 0.004	0.056 ± 0.006	0.069 ± 0.005	↑↑	↑↑↑↑	↑↑	53.753	<0.0001
Formate	0.005 ± 0.001	0.006 ± 0.001	0.004 ± 0.001	ns	↓↓↓↓	↓↓↓	31.689	<0.0001

Note: This table presents the relative concentrations of metabolites derived from 1D ^1^H NMR spectra of gastrocnemius muscle across the 2MC, 7MC, and 7ME groups. Mean values with standard deviations (mean ± SD) are provided for each metabolite concentration. Multiple group comparisons were performed using one-way ANOVA followed by Tukey’s post hoc test. Statistical significance: ns (not significant, *p* > 0.05); ↓/↑ (*p* < 0.05); ↓↓/↑↑ (*p* < 0.01); ↓↓↓/↑↑↑ (*p* < 0.001); ↓↓↓↓/↑↑↑↑ (*p* < 0.0001). Upward (↑) and downward (↓) arrows indicate an increase or decrease in the relative concentration of metabolites in pairwise comparisons.

## Data Availability

The raw data supporting the conclusions of this article will be made available by the authors on request.

## Supplementary Materials

The following supporting information can be downloaded at https://www.mdpi.com/article/10.3390/molecules30092003/s1. Figure S1: Representative 2D ^1^H-^13^C HSQC spectrum of aqueous metabolites extracted from gastrocnemius muscle of SAMP8 mice. Figure S2: Representative 2D ^1^H-^1^H TOCSY spectrum of aqueous metabolites extracted from gastrocnemius muscle of SAMP8 mice. Table S1: Resonance assignments based on 1D ^1^H NMR spectra of aqueous metabolites extracted from the gastrocnemius muscle of SAMP8 mice. Table S2: Significantly altered metabolic pathways identified from pairwise comparisons between groups.

## References

[1] Diz J.B., Leopoldino A.A., Moreira B.S., Henschke N., Dias R.C., Pereira L.S., Oliveira V.C. (2017). Prevalence of sarcopenia in older Brazilians: A systematic review and meta-analysis. Geriatr. Gerontol. Int..

[2] Baumgartner R.N., Koehler K.M., Gallagher D., Romero L., Heymsfield S.B., Ross R.R., Garry P.J., Lindeman R.D. (1998). Epidemiology of sarcopenia among the elderly in New Mexico. Am. J. Epidemiol..

[3] Hunter G.R., Singh H., Carter S.J., Bryan D.R., Fisher G. (2019). Sarcopenia and its implications for metabolic health. J. Obes..

[4] Damluji A.A., Alfaraidhy M., AlHajri N., Rohant N.N., Kumar M., Al Malouf C., Bahrainy S., Ji Kwak M., Batchelor W.B., Forman D.E. (2023). Sarcopenia and cardiovascular diseases. Circulation.

[5] Yang J., Cao R.Y., Li Q., Zhu F. (2018). Muscle atrophy in cancer. Muscle Atrophy.

[6] Fujiwara M., Ferdousi F., Isoda H. (2023). Investigation into Molecular Brain Aging in Senescence-Accelerated Mouse (SAM) Model Employing Whole Transcriptomic Analysis in Search of Potential Molecular Targets for Therapeutic Interventions. Int. J. Mol. Sci..

[7] Eckert G.P., Schiborr C., Hagl S., Abdel-Kader R., Müller W.E., Rimbach G., Frank J. (2013). Curcumin prevents mitochondrial dysfunction in the brain of the senescence-accelerated mouse-prone 8. Neurochem. Int..

[8] Wang X., Liu R., Wei C., Xu M., Li Y. (2022). Exogenous Nucleotides Improved the Oxidative Stress and Sirt-1 Protein Level of Brown Adipose Tissue on Senescence-Accelerated Mouse Prone-8 (SAMP8) Mice. Nutrients.

[9] Zeng Y., Wang P.H., Zhang M., Du J.R. (2016). Aging-related renal injury and inflammation are associated with downregulation of Klotho and induction of RIG-I/NF-κB signaling pathway in senescence-accelerated mice. Aging Clin. Exp. Res..

[10] Bernstein L.R., Mackenzie A.C., Kraemer D.C., Morley J.E., Farr S., Chaffin C.L., Merchenthaler I. (2014). Shortened estrous cycle length, increased FSH levels, FSH variance, oocyte spindle aberrations, and early declining fertility in aging senescence-accelerated mouse prone-8 (SAMP8) mice: Concomitant characteristics of human midlife female reproductive aging. Endocrinology.

[11] Karuppagounder V., Arumugam S., Babu S.S., Palaniyandi S.S., Watanabe K., Cooke J.P., Thandavarayan R.A. (2017). The senescence accelerated mouse prone 8 (SAMP8): A novel murine model for cardiac aging. Ageing Res. Rev..

[12] Nishikawa T., Takahashi J.A., Matsushita T., Ohnishi K., Higuchi K., Hashimoto N., Hosokawa M. (2000). Tubular aggregates in the skeletal muscle of the senescence-accelerated mouse; SAM. Mech. Ageing Dev..

[13] Derave W., Eijnde B.O., Ramaekers M., Hespel P. (2005). Soleus muscles of SAMP8 mice provide an accelerated model of skeletal muscle senescence. Exp. Gerontol..

[14] Guo A.Y., Leung K.S., Siu P.M., Qin J.H., Chow S.K., Qin L., Li C.Y., Cheung W.H. (2015). Muscle mass, structural and functional investigations of senescence-accelerated mouse P8 (SAMP8). Exp. Anim..

[15] Melov S., Tarnopolsky M.A., Beckman K., Felkey K., Hubbard A. (2007). Resistance exercise reverses aging in human skeletal muscle. PLoS ONE.

[16] Böhm A., Hoffmann C., Irmler M., Schneeweiss P., Schnauder G., Sailer C., Schmid V., Hudemann J., Machann J., Schick F. (2016). TGF-β Contributes to Impaired Exercise Response by Suppression of Mitochondrial Key Regulators in Skeletal Muscle. Diabetes.

[17] Fan J., Yang X., Li J., Shu Z., Dai J., Liu X., Li B., Jia S., Kou X., Yang Y. (2017). Spermidine coupled with exercise rescues skeletal muscle atrophy from D-gal-induced aging rats through enhanced autophagy and reduced apoptosis via AMPK-FOXO3a signal pathway. Oncotarget.

[18] Cunha T.F., Bacurau A.V., Moreira J.B., Paixão N.A., Campos J.C., Ferreira J.C., Leal M.L., Negrão C.E., Moriscot A.S., Wisløff U. (2012). Exercise training prevents oxidative stress and ubiquitin-proteasome system overactivity and reverse skeletal muscle atrophy in heart failure. PLoS ONE.

[19] Slavin M.B., Khemraj P., Hood D.A. (2024). Exercise, mitochondrial dysfunction and inflammasomes in skeletal muscle. Biomed. J..

[20] Steinbacher P., Eckl P. (2015). Impact of oxidative stress on exercising skeletal muscle. Biomolecules.

[21] Theilen N.T., Kunkel G.H., Tyagi S.C. (2017). The Role of Exercise and TFAM in Preventing Skeletal Muscle Atrophy. J. Cell Physiol..

[22] Sun S., Ma S., Cai Y., Wang S., Ren J., Yang Y., Ping J., Wang X., Zhang Y., Yan H. (2023). A single-cell transcriptomic atlas of exercise-induced anti-inflammatory and geroprotective effects across the body. Innovation.

[23] Booth F.W., Roberts C.K., Laye M.J. (2012). Lack of exercise is a major cause of chronic diseases. Compr. Physiol..

[24] Lazarus N.R., Harridge S.D. (2010). Exercise, physiological function, and the selection of participants for aging research. J. Gerontol. Ser. A Biomed. Sci. Med..

[25] Gremeaux V., Gayda M., Lepers R., Sosner P., Juneau M., Nigam A. (2012). Exercise and longevity. Maturitas.

[26] McPhee J.S., French D.P., Jackson D., Nazroo J., Pendleton N., Degens H. (2016). Physical activity in older age: Perspectives for healthy ageing and frailty. Biogerontology.

[27] Shur N., Creedon L., Skirrow S., Atherton P., MacDonald I., Lund J., Greenhaff P. (2021). Age-related changes in muscle architecture and metabolism in humans: The likely contribution of physical inactivity to age-related functional decline. Ageing Res. Rev..

[28] Shan S., Hoffman J.M. (2025). Serine metabolism in aging and age-related diseases. GeroScience.

[29] Tsoi B.M., Beckhouse A.G., Gelling C.L., Raftery M.J., Chiu J., Tsoi A.M., Lauterbach L., Rogers P.J., Higgins V.J., Dawes I.W. (2009). Essential role of one-carbon metabolism and Gcn4p and Bas1p transcriptional regulators during adaptation to anaerobic growth of Saccharomyces cerevisiae. J. Biol. Chem..

[30] Eller L., Wang L., Gok M.O., Hocaoglu H., Qin S., Gupta P., Sieber M.H. (2025). GSK3 coordinately regulates mitochondrial activity and nucleotide metabolism in quiescent oocytes. Biol. Open.

[31] Gonzalez-Freire M., Adelnia F., Moaddel R., Ferrucci L. (2018). Searching for a mitochondrial root to the decline in muscle function with ageing. J. Cachexia Sarcopenia Muscle.

[32] Cameron J.M., Maj M.C., Levandovskiy V., MacKay N., Shelton G.D., Robinson B.H. (2007). Identification of a canine model of pyruvate dehydrogenase phosphatase 1 deficiency. Mol. Genet. Metab..

[33] DeBalsi K.L., Wong K.E., Koves T.R., Slentz D.H., Seiler S.E., Wittmann A.H., Ilkayeva O.R., Stevens R.D., Perry C.G., Lark D.S. (2014). Targeted metabolomics connects thioredoxin-interacting protein (TXNIP) to mitochondrial fuel selection and regulation of specific oxidoreductase enzymes in skeletal muscle. J. Biol. Chem..

[34] Lian L.Y., Al-Helal M., Roslaini A.M., Fisher N., Bray P.G., Ward S.A., Biagini G.A. (2009). Glycerol: An unexpected major metabolite of energy metabolism by the human malaria parasite. Malar. J..

[35] Kernec F., Le Tallec N., Nadal L., Bégué J.M., Le Rumeur E. (1996). Phosphocreatine synthesis by isolated rat skeletal muscle mitochondria is not dependent upon external ADP: A 31P NMR study. Biochem. Biophys. Res. Commun..

[36] Gualano B., Rawson E.S., Candow D.G., Chilibeck P.D. (2016). Creatine supplementation in the aging population: Effects on skeletal muscle, bone and brain. Amino Acids.

[37] Martino M.R., Habibi M., Ferguson D., Brookheart R.T., Thyfault J.P., Meyer G.A., Lantier L., Hughey C.C., Finck B.N. (2024). Disruption of hepatic mitochondrial pyruvate and amino acid metabolism impairs gluconeogenesis and endurance exercise capacity in mice. Am. J. Physiol. Endocrinol. Metab..

[38] Pardridge W.M., Davidson M.B. (1979). Alanine metabolism in skeletal muscle in tissue culture. Biochim. Biophys. Acta.

[39] Okun J.G., Rusu P.M., Chan A.Y., Wu Y., Yap Y.W., Sharkie T., Schumacher J., Schmidt K.V., Roberts-Thomson K.M., Russell R.D. (2021). Liver alanine catabolism promotes skeletal muscle atrophy and hyperglycaemia in type 2 diabetes. Nat. Metab..

[40] Martino M.R., Gutiérrez-Aguilar M., Yiew N.K.H., Lutkewitte A.J., Singer J.M., McCommis K.S., Ferguson D., Liss K.H.H., Yoshino J., Renkemeyer M.K. (2022). Silencing alanine transaminase 2 in diabetic liver attenuates hyperglycemia by reducing gluconeogenesis from amino acids. Cell Rep..

[41] Holeček M. (2023). Aspartic Acid in Health and Disease. Nutrients.

[42] McNair L.M., Andersen J.V., Waagepetersen H.S. (2023). Stable isotope tracing reveals disturbed cellular energy and glutamate metabolism in hippocampal slices of aged male mice. Neurochem. Int..

[43] Kimoloi S. (2023). Exercise-induced fumarate accumulation: A potential mediator of mitochondrial biogenesis in mammalian skeletal muscle. Int. J. Sport Exerc. Health Res..

[44] Henderson G.C., Horning M.A., Lehman S.L., Wolfel E.E., Bergman B.C., Brooks G.A. (2004). Pyruvate shuttling during rest and exercise before and after endurance training in men. J. Appl. Physiol..

[45] Consitt L.A., Saxena G., Saneda A., Houmard J.A. (2016). Age-related impairments in skeletal muscle PDH phosphorylation and plasma lactate are indicative of metabolic inflexibility and the effects of exercise training. Am. J. Physiol.-Endocrinol. Metab..

[46] Huh J.Y., Mougios V., Kabasakalis A., Fatouros I., Siopi A., Douroudos I.I., Filippaios A., Panagiotou G., Park K.H., Mantzoros C.S. (2014). Exercise-induced irisin secretion is independent of age or fitness level and increased irisin may directly modulate muscle metabolism through AMPK activation. J. Clin. Endocrinol. Metab..

[47] Dutka T.L., Lamboley C.R., Murphy R.M., Lamb G.D. (2014). Acute effects of taurine on sarcoplasmic reticulum Ca2+ accumulation and contractility in human type I and type II skeletal muscle fibers. J. Appl. Physiol..

[48] Seidel U., Huebbe P., Rimbach G. (2019). Taurine: A regulator of cellular redox homeostasis and skeletal muscle function. Mol. Nutr. Food Res..

[49] Wang Y., Xu T., Zhao H., Gu C., Li Z. (2022). Effect of taurine in muscle damage markers and inflammatory cytokines in running exercise. Front. Physiol..

[50] Ma Y., Maruta H., Sun B., Wang C., Isono C., Yamashita H. (2021). Effects of long-term taurine supplementation on age-related changes in skeletal muscle function of Sprague-Dawley rats. Amino Acids..

[51] Liu H.W., Chang Y.C., Chan Y.C., Hu S.H., Liu M.Y., Chang S.J. (2020). Dysregulations of mitochondrial quality control and autophagic flux at an early age lead to progression of sarcopenia in SAMP8 mice. Biogerontology..

[52] Chen L.H., Chang S.S., Chang H.Y., Wu C.H., Pan C.H., Chang C.C., Chan C.H., Huang H.Y. (2022). Probiotic supplementation attenuates age-related sarcopenia via the gut–muscle axis in SAMP8 mice. J. Cachexia Sarcopenia Muscle.

[53] Aoki K., Konno M., Honda K., Abe T., Nagata T., Takehara M., Sugasawa T., Takekoshi K., Ohmori H. (2020). Habitual aerobic exercise diminishes the effects of sarcopenia in senescence-accelerated mice Prone8 model. Geriatrics.

[54] Andreani C., Bartolacci C., Guescini M., Battistelli M., Stocchi V., Orlando F., Provinciali M., Amici A., Marchini C., Tiano L. (2018). Combination of coenzyme Q10 intake and moderate physical activity counteracts mitochondrial dysfunctions in a SAMP8 mouse model. Oxidative Med. Cell. Longev..

[55] Park K.H., Brotto L., Lehoang O., Brotto M., Ma J., Zhao X. (2012). Ex vivo assessment of contractility, fatigability and alternans in isolated skeletal muscles. J. Vis. Exp..

[56] Le Gall G. (2015). Sample collection and preparation of biofluids and extracts for NMR spectroscopy. Methods Mol. Biol..

[57] Beckonert O., Keun H.C., Ebbels T.M., Bundy J., Holmes E., Lindon J.C., Nicholson J.K. (2007). Metabolic profiling, metabolomic and metabonomic procedures for NMR spectroscopy of urine, plasma, serum and tissue extracts. Nat. Protoc..

[58] Mckay R.T. (2011). How the 1D-NOESY suppresses solvent signal in metabonomics NMR spectroscopy: An examination of the pulse sequence components and evolution. Concepts Magn. Reson. Part A.

[59] Vignoli A., Ghini V., Meoni G., Licari C., Takis P.G., Tenori L., Turano P., Luchinat C. (2019). High-Throughput Metabolomics by 1D NMR. Angew. Chem. Int. Ed..

[60] Bingol K., Bruschweiler-Li L., Li D.W., Brüschweiler R. (2014). Customized metabolomics database for the analysis of NMR ^1^H-^1^H TOCSY and ^13^C-^1^H HSQC-TOCSY spectra of complex mixtures. Anal. Chem..

[61] Pang Z., Zhou G., Ewald J., Chang L., Hacariz O., Basu N., Xia J. (2022). Using MetaboAnalyst 5.0 for LC-HRMS spectra processing, multi-omics integration and covariate adjustment of global metabolomics data. Nat. Protoc..

