# Supplementary material for: Exercise Attenuates Skeletal Muscle Atrophy in Senescent SAMP8 Mice: Metabolic Insights from NMR-Based Metabolomics
Source: Molecules. 2025 Apr 30;30(9):2003. doi: 10.3390/molecules30092003 (PMC12073869; doi:10.3390/molecules30092003)
Supplement: Supplementary file 1 [file molecules-30-02003-s001.zip › molecules-3551391-supplementary.pdf]

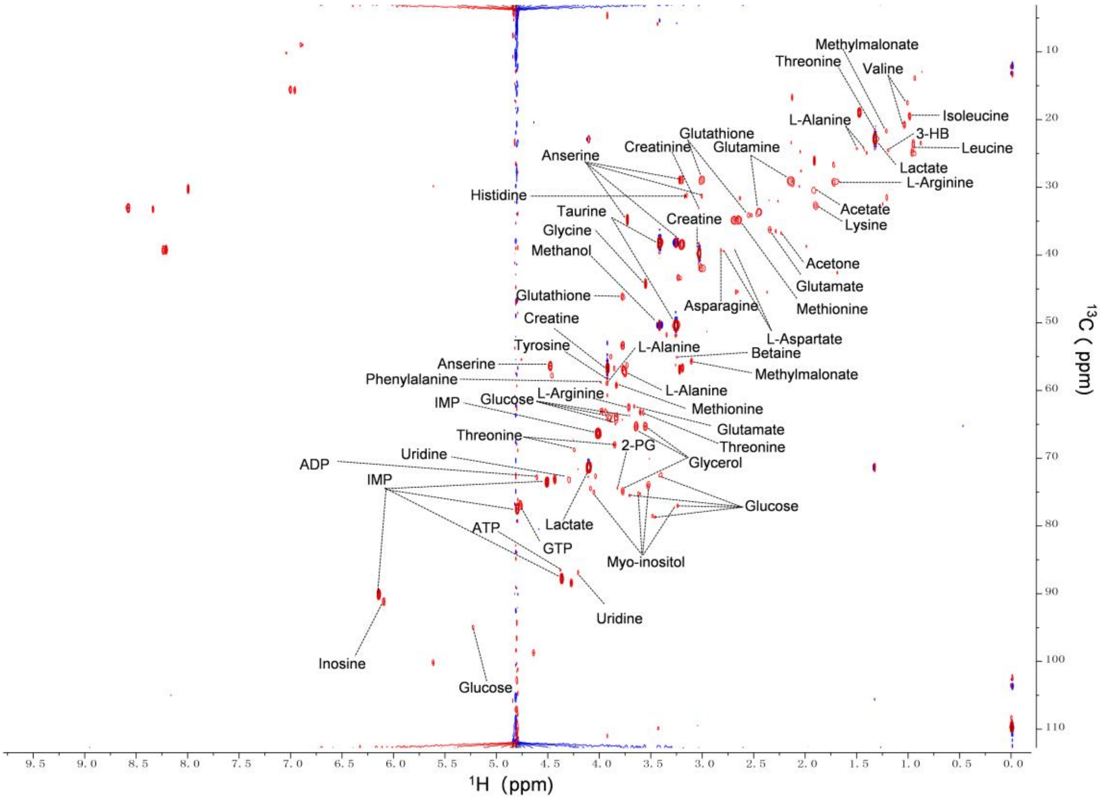

Figure S1. Representative 2D ^{1}H-^{13}C HSQC spectrum of aqueous metabolites extracted from gastrocnemius muscle of SAMP8 mice. The spectrum was recorded on an 850 MHz NMR spectrometer (pH 7.4, 298 K). Detailed acquisition parameters, including pulse sequence, spectral width, relaxation delay, and data matrix dimensions, are provided in the main text (Section 5.6).

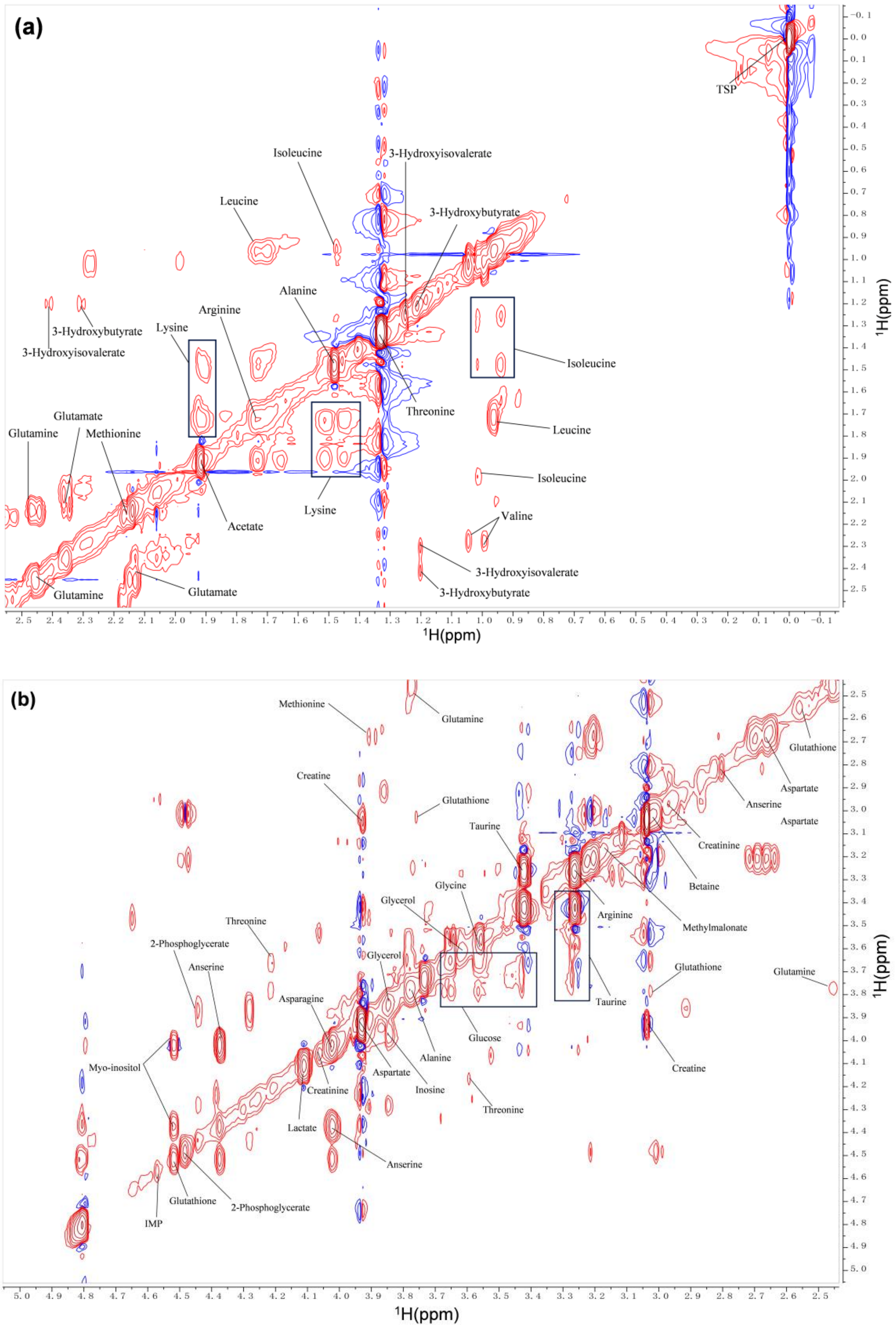
(a)
TSP
Isoleucine
3-Hydroxyisovalerate
Leucine
3-Hydroxybutyrate
Alanine
Arginine
Lysine
3-Hydroxybutyrate
3-Hydroxyisovalerate
Isoleucine
Threonine
Glutamate
Glutamine
Methionine
Leucine
Isoleucine
Acetate
Lysine
Valine
3-Hydroxyisovalerate
3-Hydroxybutyrate
Glutamine
Glutamate
^{1}H(ppm)
^{1}H(ppm)
(b)
Methionine
Glutamine
Glutathione
Creatine
Glutathione
Aspartate
Anserine
Aspartate
Taurine
Creatinine
Betaine
Glycine
Glycerol
Arginine
Methylmalonate
Threonine
2-Phosphoglycerate
Glycerol
Anserine
Glutathione
Glutamine
Asparagine
Taurine
Glucose
Myo-inositol
Alanine
Aspartate
Creatine
Inosine
Creatinine
Threonine
Lactate
Anserine
Glutathione
2-Phosphoglycerate
IMP
^{1}H(ppm)
^{1}H(ppm)

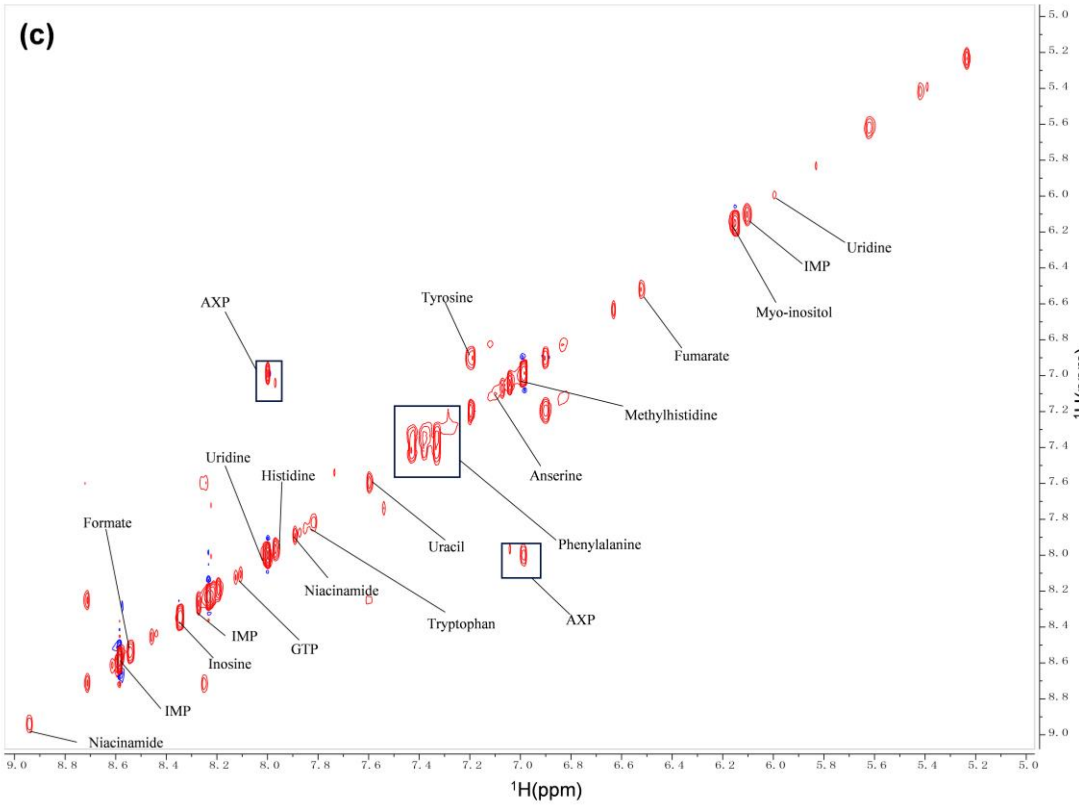

Figure S2. Representative 2D ^{1}H-^{1}H TOCSY spectrum of aqueous metabolites extracted from gastrocnemius muscle of SAMP8 mice. The spectrum was recorded on an 850 MHz NMR spectrometer (pH 7.4, 298 K). (a) 0.0-2.5 ppm; (b) 2.5-5.0 ppm; (c) 5.0-9.0 ppm. Detailed acquisition parameters, including pulse sequence, spectral width, relaxation delay, and data matrix dimensions, are provided in the main text (Section 5.6).

Table S1 Resonance assignments based on 1D ^{1}H NMR spectra of aqueous metabolites extracted from the gastrocnemius muscle of SAMP8 mice.

Metabolite	δ ^{1}H(ppm) and multiplicity	Moieties
Leucine	0.96(d), 0.97(d), 1.69(m), 1.70(m), 1.73(m), 3.73(m)	α-CH_3, α-CH_3, γ-CH, α-CH_3, β-CH_2, α-CH
Isoleucine	0.94(t), 1.01(d), 1.21(m), 1.42(m), 2.00(m), 3.67(d)	δ-CH_3, γ-CH_3, half γ-CH_2, half γ-CH_2, β-CH, α-CH
Valine	0.99(d), 1.05(d), 2.26(m), 3.60(d)	γ-CH_3, γ-CH_3, β-CH, α-CH
Ethanol	1.17(t), 3.65(q)	δ-CH_3, CH_2
3-Hydroxybutyrate	1.23(d), 2.29(dd), 2.39(dd), 4.14(m)	γ-CH_3, half α-CH_2, half α-CH_2, β-CH
3-Hydroxyisovalerate	1.25(s), 2.35(s)	γ-CH_3, α-CH_2
Methylmalonate	1.23(d), 3.16(q)	$^{5}CH_3$, ^{4}CH
Alanine	1.47(d), 3.78(q)	β-CH_3, α-CH
Lysine	1.89(m), 1.92(m), 3.02(t), 3.75(t)	γ-CH_2, half γ-CH_2, δ-CH_2, β-CH_2, ε-CH_2, α-CH
Acetate	1.91(s)	CH_3
Arginine	1.69(m), 1.91(m), 3.24(t), 3.75(t)	α-CH_2, β-CH_2, N-CH_2, N-CH
Methionine	2.16(m), 2.64(t), 3.85(dd)	δ-CH3, γ-CH2, α-CH
Glutamate	2.08(m), 2.12(m), 2.34(m), 2.37(m), 3.75(m)	half β-CH_2, half β-CH_2, half γ-CH_2, half γ-CH_2, α-CH
Glutamine	2.13(m), 2.45(m), 3.77(t)	γ-CH_2, β-CH_2, α-CH
Glutathione	2.15(m), 2.55(m), 2.96(m), 3.77(m), 4.56(m)	β-CH_2, γ-CH_2, CH_2-SH, α-CH&CH_2-NH, CH-NH
Anserine	4.02(s), 8.28(s), 6.76(s), 3.14(d), 4.35(d), 7.14(d), 2.79(t), 2.86(t)	CH_3, CH_2, CH, CH-NH, N-CH, NH_2
Aspartate	2.68(dd), 2.81(dd), 3.90(dd)	β-CH_2, α-CH
Asparagine	2.84(dd), 2.94(dd), 4.00(dd)	half β-CH, half β-CH, α-CH
Creatine	3.04(s), 3.93(s)	N-CH_3, α-CH_2
Betaine	3.10(s), 3.58(s)	$^{1,3,4}CH_3$, $^{5}CH_2$
Taurine	3.24(t),3.41(t)	^{1} CH_2, ^{2} CH_2
Glycine	3.57(s)	α-CH_2
Threonine	1.31(d), 3.59(d), 4.25(m)	γ-CH_2, β-CH
Glycerol	3.6(d), 3.85(m)	α-CH_2, γ-CH_2, β-CH
2-Phosphoglycerate	3.81(q), 3.88(q), 4.49(m),	γ-CH_2, β-CH
Creatinine	3.03(s), 4.05(d)	CH_3, N-CH
Lactate	1.33(d), 4.11(q)	β-CH_3, α-CH
Myo-inositol	3.28(t), 3.53(dd), 3.63(t), 4.07(t)	^{2} CH, ^{4,6} CH, ^{1,3} CH, ^{5} CH
IMP	4.02(m), 4.37(m), 4.52(q), 6.15(d), 8.23(s)， 8.58(s)	CH_2, CH, CH-OH, N-CH, N-CH-N

Glucose	β(3.24(dd), 3.48(t), 3.90(dd)), α(3.54(dd), 3.71(t), 3.72(dd), 3.83(m))	β(H_2, H_3, H_5), α(H_2, H_3, H_6)
Uracil	6.18(d), 7.53(d)	CH, NH-CH
GTP	5.92 (d), 8.1 (s)	CH, CH
Inosine	3.83(d), 3.84(d), 6.1(d), 8.23(s), 8.35(s)	CH_2-OH, CH, CH-OH, O-CH-N, CH-NH, N-CH-N
Fumarate	6.51(s)	CH
Tyrosine	3.05(dd), 3.19(dd), 6.92(d), 7.19(d)	half β-CH_2,half β-CH_2, β-CH, α-CH
Methylhistidine	8.03(s), 7.01(s), 3.95(dd)	N-CH-N, N-CH, CH-NH_2
Phenylalanine	3.12(dd), 3.30(dd), 3.99(dd), 7.33(d), 7.37(t), 7.43(t)	α-CH, half β-CH_2, half β-CH_2, α-CH, β-CH, γ-CH
Niacinamide	7.81(m), 7.57(m), 8.59(dd), 8.89(dd)	CH, CH-N
Tryptophan	7.73(d), 7.54(d), 7.32(s)	CH, CH, CH-NH
Uridine	7.87(d), 5.92(d), 5.90(d)	N-CH, CH-N, CH
Histidine	7.06(s), 7.85(s)	^{5}CH, ^{2}CH
Adenine	8.01(s)	N-CH-N, N-CH-NH
AXP	6.14(d), 8.27(s), 8.58(s)	NH_2, δ-CH, ^{2}CH
Formate	8.46(s)	CH

Note: s, singlet; d, doublet; t, triplet; q, quartet; m, multiplet; dd, doublet of doublets. (IMP, Hypoxanthine nucleotides; GTP, guanosine triphosphate; AXP, adenine mono/di/triphosphate). The underlined chemical shift values represent the characteristic peaks of each metabolite, which were utilized for quantifying relative metabolite concentrations based on their NMR integrals. These selected peaks ensure accuracy and consistency in metabolite concentration calculations.

Table S2. Significantly altered metabolic pathways identified from pairwise comparisons between groups.

ID	Metabolic Pathway	7MC *vs.* 2MC	7ME *vs.* 7MC
a	Alanine, aspartate and glutamate metabolism	✓	✓
b	Pyruvate metabolism	✓	✓
e	Starch and sucrose metabolism	✓	
c	Glycine, serine and threonine metabolism	✓	✓
f	Glycerolipid metabolism	✓	
d	Taurine and hypotaurine metabolism	✓	✓
g	Phenylalanine, tyrosine and tryptophan biosynthesis	✓	
h	Phenylalanine metabolism	✓	
i	Histidine metabolism	✓	
